# Lifestyle intervention to improve quality of life and prevent weight gain after renal transplantation: Design of the Active Care after Transplantation (ACT) randomized controlled trial

**DOI:** 10.1186/s12882-017-0709-0

**Published:** 2017-09-15

**Authors:** Gerald Klaassen, Dorien M. Zelle, Gerjan J. Navis, Desie Dijkema, Frederike J. Bemelman, Stephan J.L. Bakker, Eva Corpeleijn

**Affiliations:** 1 Department of Internal Medicine, division of Nephrology, University Medical Center Groningen, University of Groningen, Groningen, the Netherlands; 2 Department of Dietetics, University Medical Center Groningen, Groningen, the Netherlands; 3 Department of Nephrology, Amsterdam Medical Center, University of Amsterdam, Amsterdam, the Netherlands; 4 Department of Epidemiology, University Medical Center Groningen, University of Groningen, Groningen, the Netherlands

**Keywords:** Kidney, Physical activity, Cardiovascular, Exercise, Diet, Body weight

## Abstract

**Background:**

Low physical activity and reduced physical functioning are common after renal transplantation, resulting in a reduced quality of life. Another common post-transplantation complication is poor cardio-metabolic health, which plays a main role in long-term outcomes in renal transplant recipients (RTR). It is increasingly recognized that weight gain in the first year after transplantation, especially an increase in fat mass, is a highly common contributor to cardio-metabolic risk. The aim of this study is to compare the outcomes of usual care to the effects of exercise alone, and exercise combined with dietary counseling, on physical functioning, quality of life and post-transplantation weight gain in RTR.

**Methods:**

The Active Care after Transplantation study is a multicenter randomized controlled trial with three arms in which RTR from 3 Dutch hospitals are randomized within the first year after transplantation to usual care, to exercise intervention (3 months supervised exercise 2 times per week followed by 12 months active follow-up), or to an exercise + diet intervention, consisting of the exercise training with additional dietary counseling (12 sessions over 15 months by a renal dietician). In total, 219 participants (73 per group) will be recruited. The primary outcome is the subdomain physical functioning of quality of life, (SF-36 PF). Secondary outcomes include other evaluations of quality of life (SF-36, KDQOL-SF, EQ-5D), objective measures of physical functioning (aerobic capacity and muscle strength), level of physical activity, gain in adiposity (body fat percentage by bio-electrical impedance assessment, BMI, waist circumference), and cardiometabolic risk factors (blood pressure, lipids, glucose metabolism). Furthermore, data on renal function, medical history, medication, psychological factors (motivation, kinesiophobia, coping style), nutrition knowledge, nutrition intake, nutrition status, fatigue, work participation, process evaluation and cost-effectiveness are collected.

**Discussion:**

Evidence on the effectiveness of an exercise intervention, or an exercise + diet intervention on physical functioning, weight gain and cardiometabolic health in RTR is currently lacking. The outcomes of the present study may help to guide future evidence-based lifestyle care after renal transplantation.

**Trial registration:**

Number: NCT01047410.

**Electronic supplementary material:**

The online version of this article (10.1186/s12882-017-0709-0) contains supplementary material, which is available to authorized users.

## Background

Low physical activity, reduced physical fitness and reduced physical functioning are common after renal transplantation [1]. Physical activity levels decline already in the early stages of chronic kidney disease, and reach a nadir at end-stage renal disease [1]. This low physical activity aggravates the catabolic state that is common in patients with renal disease, which leads to deconditioning and reduced physical functioning [1]. Spontaneous recovery of physical activity level after transplantation is modest, and limited physical functioning remains common [1]. Likewise, low cardiorespiratory fitness and muscle weakness are also common after renal transplantation [1]. This may be improved with exercise and physical activity, however, RTR experience barriers to engage in exercise and physical activity such as fear of movement, lack of motivation, and fatigue [1, 2].

**Fig. 1:**
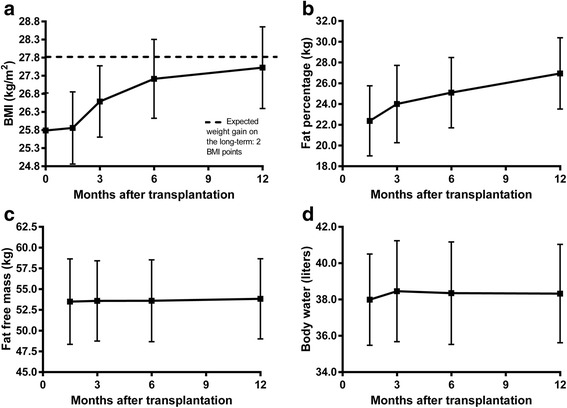
Changes in body weight and body composition after renal transplantation. (**a**: BMI, **b**: body fat percentage, **c**: fat-free mass, and **d**: whole body water content) Reprinted from “The role of diet and physical activity in post-transplant weight gain after renal transplantation” by D.M. Zelle, 2013, Clinical Transplantation, 27: E484-E490. Reprinted with permission

The incidence and prevalence of cardiovascular disease is estimated to be 4 to 6 times higher in RTR compared to the general population [3]. Various factors may explain poor post-transplantation health in RTR, including the legacy of end-organ damage due to the prior medical history (e.g. cardiovascular damage), that may not be fully reversible by restoring renal function. Importantly, the common substantial post-transplantation weight gain, which is primarily due to an increase in body fat mass, is a major trigger for the poor cardio-metabolic profile in the RTR, including post-transplant diabetes and the metabolic syndrome [4–9]. Furthermore, post-transplantation BMI is an important risk factor for graft failure and premature death [10–13]. A poor diet post-transplantation may be related to pre-transplantation dietary prescriptions such as low intake of vegetables to avoid hyperkalemia and a high intake of energy rich drinks to ensure adequate energy intake [14, 15]. Additionally, glucocorticoid use after transplantation often promotes overeating, so a healthy diet without an excess of calories is frequently required [16]. Data from a previous study showed that RTR gain an average of 5.7 ± 5.0 Kg of body weight in the first year after transplantation (Fig. 1) [4]. Interestingly, the rise in body weight was related to low physical activity and poor diet rather than increased energy intake [4].

From the above, it may be clear that both physical activity and diet are important targets for lifestyle intervention in RTR. The importance of physical activity and exercise for health outcomes is well-acknowledged in the general population [17]. In RTR however, high level evidence on the effects of physical activity and exercise on physical functioning and cardiometabolic health is limited and comes from few small RCTs [18, 19]. The available studies however, including single group interventions, show promising results. Dietary intervention after renal transplantation was shown to reduce body weight and improve lipid profile [20], whereas intensive lifestyle intervention improved glucose metabolism [21]. Importantly, it was also shown that a supervised exercise program lead to similar improvements in muscle strength and aerobic capacity in RTR compared to healthy individuals [22].The first year after transplantation may provide a window of opportunity to improve physical functioning and quality of life, and to prevent post-transplantation weight gain and its cardiometabolic consequences. Assuming that muscle disuse is a major factor in muscle weakness and low aerobic capacity in RTR, exercise rehabilitation and increased daily physical activity may be of great benefit to this patient population. For physical activity and exercise to result in a favourable shift from a catabolic to an anabolic state, proper consideration of nutritional status, in particular energy and protein intake, and metabolic status is necessary. Furthermore, dietary counseling directed at restoring a healthy eating pattern may prevent post-transplant weight gain and improve dietary quality.

The Active Care after Transplantation (ACT) study is a pragmatic randomized controlled trial designed to evaluate the effects of an exercise intervention, and an exercise + dietary intervention, on physical functioning and quality of life after renal transplantation. In addition, detailed measures of physical fitness, level of physical activity, gain in adiposity, renal function, and cardiometabolic profile will be investigated, as well as success factors for lifestyle change in RTR. A health economics evaluation is included.

## Methods/design

**Fig. 2:**
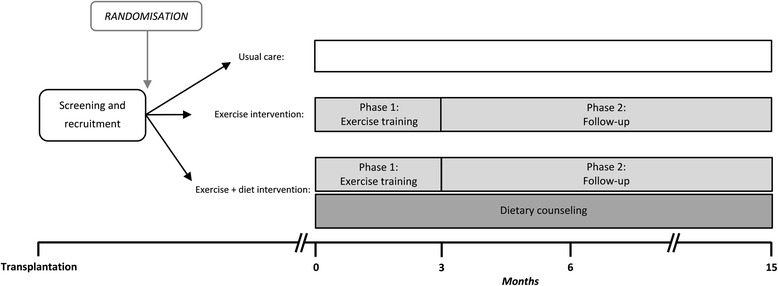
Flowchart of the ACT trial

Active Care After Transplantation (ACT) is a multicenter pragmatic randomized controlled trial in renal transplant patients in the Netherlands, registered on www.clinicaltrials.gov as NCT01047410. The ACT trial has three study arms: usual care versus exercise intervention versus exercise + diet intervention (Fig. 2). It is designed according to the principles of a pragmatic RCT to allow evaluation of the intervention in real-life care facilities. Details are described below according to the CONSORT statement for parallel randomized trials and the extension of the CONSORT statement for pragmatic trials [23–25]. The study is performed according to the declaration of Helsinki and ethical approval is obtained from the University Medical Center Groningen Medical Ethics committee (NL49084.042.14). Adverse- and serious adverse events data are collected and closely monitored to ensure the ongoing safety of the participants. Both are reported in a standardized Adverse Events Report Form. Exemption to report adverse events is given for common renal transplant related co-morbidities, such as cardiovascular events, urinary tract infections, viral infections, pneumonia, urine retention, rejection, cardiac decompensation, hydronephrosis, nephrostomy catheter, lymphocele, hypertension, dyslipidemia, diabetes, post-transplantation lymphoma, cerebrovascular accidents, meningitis, and skin cancer.

### Eligibility criteria

The target population for the study are adult RTR, recruited during routine post-transplantation care shortly, but at least within the first year after transplantation. Inclusion criteria are: written informed consent; age > 18 years; < 1 year after transplantation; approval for participation based on a general medical evaluation by their nephrologist. Exclusion criteria included the following: multiple organ transplantation; psychopathology or serious cognitive impairment; physical or clinical limitations that make participation impossible; pregnancy, negative screening verdict by the nephrologist or cardiologist. Participants receive a small financial reimbursement (10 Euro) for participation in the final follow-up measurement at 15 months. Participation in the exercise training sessions and dietary care are financed by research funds, and participants’ travel expenses are reimbursed. Pre-defined reasons to discontinue participation in the study included pregnancy and conversion to dialysis. Furthermore, reasons to discontinue participation are: changes in ECG during maximal aerobic capacity testing, systolic blood pressure > 250 mmHg, diastolic blood pressure > 120 mmHg or blood pressure decline of > 20 mmHg during maximal aerobic capacity testing, and severe cardio-pulmonary complaints during the exercise trainings sessions.

### Screening, recruitment and randomization

All RTR patients are screened for eligibility during the post-transplantation recovery period, so within 7–10 days after surgery, by the nephrologist. Eligible RTR are invited to participate, informed about the study protocol and if agreed are asked to participate and sign written informed consent. In case of non-Dutch or non-English speaking patients, interpreters are used.

In total, 219 participants (73 per group) will be recruited. The sample size is based on the expected improvement on the physical functioning scale of quality of life of 13.8 points in the intervention group and 8.8 points in usual care (α=0.05, β= 0.80, SD = 11.0) [26]. A difference of 5.0 points between the standard effect is considered a relevant effect. This results in a required number of 57 RTR per group. Taking into account a potential dropout rate of 15% and a correction for heterogeneity between centers of 10%, this results in a total required number of 73 RTR per group (*N* = 219 in total). Randomization to one of the three arms is computerized using the ‘PROC SURVEYSELECT’ function in the software program SAS (Cary, North Carolina, USA). It is performed by an independent data management company hosting the data entry facility, who are not involved in conducting the study itself (Nefrovisie foundation, Utrecht, the Netherlands). Participants are randomized by block randomization in groups of 6–9 for rehabilitation centers and in groups of 3 for physiotherapy practices.

### Setting and participants

Recruitment takes place in three Dutch hospitals with good geographical coverage, in the north (University Medical Center Groningen), west (Amsterdam Medical Center) and south (Maastricht University Medical Center) of The Netherlands. The exercise training is offered in rehabilitation centers or in local physiotherapy practices if there is no rehabilitation center within reasonable travel distance of the residence of the participant.

Delivery of the exercise training is standardised by providing protocols and the same exercise training protocol for all interventionists.

### Professionals

The interventionists of the participating centers all received personal instructions of exercise scientists on the protocols, the basic medicine of renal disease, and adverse effects of the immunosuppressive- and other medication. Furthermore, all interventionists participated in a mandatory kickoff meeting where the lifestyle counseling part of the intervention was explained and practiced, including the theoretical background (stages of change model and motivational interviewing). All participating rehabilitation centers and physiotherapy practices meet the following criteria: expertise in rehabilitation of cardiovascular or oncology patients; adequate training facilities; all the necessary fitness equipment for submaximal cycle ergometry and strength tests; and, for physiotherapy practices only, provide opportunities for participants to continue their fitness activities during the active follow-up.

Concerning the diet intervention, dieticians are required to meet the following selection criteria: experience in counseling renal patients or other patients with a serious chronic metabolic condition; educated and experienced in motivational interviewing; educated and experienced in Subjective Global Assessment of malnutrition. Lifestyle coaches had to fulfill the following selection criteria: experience in lifestyle counseling of renal patients or other patients with a serious chronic metabolic condition; educated and experienced in motivational interviewing. In most cases the lifestyle counseling is done by the physiotherapist who supervises the training.

A website dedicated to the study (in Dutch language) provides all protocols, explanations and hosts a data entry system (www.act-studie.nl).

### Usual care

Participants randomized to the usual care group receive standard post-transplant care as usual in the hospitals. Current standard nutrition care after kidney transplantation consists of 1–4 inpatient consultations by a renal dietitian during the inpatient stay (7–10 days), followed by 1 outpatient consultation. During inpatient consultations attention is paid to optimal nutritional intake, maintaining a stable body weight and muscle mass, or increasing body weight when needed. After a few months, during the outpatient follow-up, attention is paid to maintaining a stable body weight, or increasing or decreasing body weight when needed. When a participant has increased cholesterol, (un)saturated fat intake is discussed, and mono/disaccharides are discussed when a participant has increased HbA1c. Likewise, salt consumption is discussed in case of increased 24-h sodium excretion. Exercise or lifestyle counseling are not included in the standard post-transplant care in either of the participating hospitals. Participants randomized to the usual care group receive general advice to meet the standard Dutch exercise guidelines (30 min of moderate physical activity per day) and follow the Dutch Dietary guidelines.

### Exercise only intervention

The exercise intervention consists of two phases. The first phase is a 3-month exercise training program, followed by the second phase; a 12-month active follow-up with lifestyle counseling (Figure 2). Exercise training sessions are done in groups of 4–10 participants in the rehabilitation centers and with 1–6 participants in the physiotherapy centers.

The training program encompasses two exercise training sessions per week (21 exercise training sessions and 3 test sessions in total) and is focused on improving maximal muscle strength, local muscle endurance, and aerobic capacity. Each exercise training session consists of 30-min dynamic resistance training of all the major muscle groups (Additional file 1: Table S1) using resistance training machines, 30 min of aerobic training (cycle-ergometry and treadmill walking), and is concluded with a 30-min supervised sports activity or swimming, depending on the available facilities.

The dynamic resistance training includes both maximal strength training (relatively high load, fewer repetitions), and muscle endurance training (relatively low load, large number of repetitions).

Optimal resistance training load is determined using one-repetition maximum testing (1RM) for each participant. The content of the training program is in line with the American College of Sports Medicine guidelines exercise in healthy adults [27], and was previously shown to be effective in improving muscle strength and aerobic capacity in RTR [22]. The full resistance- and aerobic training program is shown in Additional files 1 and 2: Tables S1 and S2.

Maximal muscle strength (1RM) for all exercise machines (Additional file 1: Table S1) is assessed at baseline. For maximal muscle strength training, moderate loading is used (50–60% 1RM), for 2–3 sets of 8–10 repetitions. Higher loads are known to be more appropriate for maximal muscle strength, however 50–60% 1RM is used in our protocol as most participants are untrained, and RTR have increased risk of tendon ruptures. For local muscle endurance training a light-to-moderate load is used (25–35% 1RM), for 1–3 sets of 30 repetitions. All resistance exercise involved exercise machines, no free weights are used for safety reasons.

The load for the aerobic cycle training is determined based on the baseline symptom-limited graded maximal cycle ergometry test. Treadmill walking is performed at the load at which heart rate is similar to the heart rate during aerobic cycle-training. If the physiotherapists notice that a subject is training below or above the optimal intensity (e.g. in case a baseline exercise test is performed sub optimally), they are allowed to make slight adjustments to the participants’ exercise intensity.

#### Lifestyle counseling

During the active follow-up, participants are supported to continue sports activities and keep up daily physical activity by lifestyle coaches. A total of 5 lifestyle counseling consultations are scheduled during the entire exercise intervention and similar for the exercise + diet intervention, see next paragraph: at 6 weeks, 12 weeks, 16 weeks, 6 months, and at 15 months. The first two consultations are face-to-face and combined with the exercise training sessions. Consultations at 4 and 6 months are done face-to-face if possible, otherwise by phone. The final lifestyle counseling session is face-to-face together with the final test moment at 15 months. To optimize participant motivation, the lifestyle counseling is in line with the Self-Determination Theory, fostering the perceived autonomy, competence and relatedness [28–30]. Additionally, the Stages of Change model is applied, and readiness for change is assessed at the first 2 lifestyle counseling sessions to better tailor the content of the consultations to the participants' readiness to change their exercise and physical activity behavior [31]. During these lifestyle counseling sessions a motivational interviewing approach is used. During the lifestyle counseling participants receive several tools to stimulate a physically active lifestyle, including an information leaflet of the Dutch Kidney Foundation on exercise and physical activity after renal transplantation [32], a step counter to stimulate physical activity through self-monitoring, and finally, a hard-copy log book for educational and motivational purposes. The log book includes advice on exercise, finding an exercise buddy, setting personal goals, and tables to log one’s own exercise behavior.

### Exercise + diet intervention

The exercise training program in the exercise + diet intervention group is identical to that of the exercise intervention group, and additionally, participants receive dietary counseling. The dietary counseling runs throughout the entire 15 months of the study period and consists of a total of 12 counseling sessions with a renal dietician. The dietary counseling takes place in the hospital face-to-face. If this is not feasible some counseling sessions are done by phone. The dieticians are trained to use motivational interviewing techniques which they apply during the dietary counseling sessions.

Goals are set to improve nutrition status to preserve renal function, to prevent weight gain, type 2 diabetes, and hypercholesterolemia. These goals are set together with the participant to facilitate an autonomy supportive counseling climate [33]. A three-day food diary is used as input during the dietary consultations and for personal goal setting. Participants also receive a food logbook which contains information and exercises to help the participant set diet goals. During the dietary counseling sessions, special attention is given to salt intake, whole-wheat grains and fruit and vegetable intake, intake of saturated and unsaturated fat, and the use of sugar-rich products, sugar-sweetened beverages (soft drinks, sweetened dairy drinks and fruit juices) and preventing overeating. In addition, participants are reminded of their physical activity goals, and the importance of protein intake for maintaining and increasing muscle mass. In case of disease after transplantation, for example infections, the dietician assists the patient in maintaining an energy- and protein-rich diet to prevent further deterioration or decline in muscle mass.

Dietary counseling session 9 (of total 12) is a group meeting for which the participants are encouraged to bring significant others such as their partner or other supportive family members. The dietary counseling is in line with the 2006 Guidelines for a Healthy Diet by the Dutch Food and Nutrition Council [34], supplemented with diet guidelines for RTR [35] and diet guidelines for the prevention of type 2 diabetes [36] and cardiovascular risk [37].

### Immunosuppressive medication regimen

All participants receive standard immunosuppressive treatment. In the UMCG this consists of induction therapy with methylprednisolone (40 mg) and basiliximab (20 mg) on day 1 and at day 4 post-operation. This is followed by a maintenance immunosuppressive regimen of tacrolimus, mycophenolate mofetil and prednisolone, tapered to a maintenance dose of 5-10 mg per day. Standard immunosuppressive treatment in the MUMC consists of no induction therapy, methylprednisolone 125 mg per day intravenous during the first 48 h, starting perioperative, followed by a maintenance immunosuppressive regimen of mycofenolate mofetil 2dd 1000 mg, after two weeks 2dd 500 mg and tacrolimus .

Standard immunosuppressive treatment in the AMC consisted of induction therapy CD25 mAB intravenous prior to transplantation, and at day 4 post-operation. Methyl prednisolone 2 × 50 mg intravenous during the first 48 h, starting perioperative. This is followed by a maintenance immunosuppressive regimen of tacrolimus, mycophenolate mofetil and prednisolone tapered to a dose of 5–10 mg per day.

### Outcomes

The primary outcome is the subdomain physical functioning of quality of life of the SF-36. Secondary outcomes include other evaluations of quality of life (SF-36, KDQOL-SF, EQ-5D), muscle strength (Additional file 1: Table S1), aerobic capacity, physical activity, gain in adiposity (body fat percentage by bio-electrical impedance assessment, BMI, waist circumference), and cardiometabolic risk factors (blood pressure, lipids, glucose metabolism). Furthermore, data on renal function, medical history, medication, psychosocial factors (motivation, kinesiophobia, coping style), nutrition knowledge, nutrition intake, nutrition status, fatigue, work participation, process evaluation and cost-effectiveness are collected. Effectiveness of the diet intervention will be determined at 15 months. To study barriers and success factors regarding lifestyle counseling in RTR, data are gathered on psychological factors including motivation, kinesiophobia, and coping style. A comprehensive overview of the measurements in the ACT trial is shown in Table 1.

**Table 1:** Overview of measurements in the ACT trial

Outcome	Method	Time points
Quality of life		T0, T3, T6, T15
Physical functioning	SF-36 PF subscale [72, 73]	
Other subdomains and total	SF-36 [72, 73]	
Renal specific	KDQOL-SF [39]	
General	EQ-5D [69]	
Physical function		
Aerobic capacity	VO_2_peak ergometer test [74]	T0, T3, T6, T15
	Sub-maximal ergometer test	T0, T3, T6, T15
Muscle strength	1RM tests for leg extension, leg curl, chest press, close-grip pull-down	T0, T3, T6, T15
Balance and mobility	Short Physical Performance Battery [41]	T0, T15
Physical activity level	Baecke questionnaire [42]	T0, T3, T6, T15
	Step counter (Yamax SW200) [75]	T0, T3, T6, T15
Body composition		
Weight, Height, WC, HC	WHO guidelines [76]	T0, T3, T6, T15
Nutritional status (undernutrition)	SGA [77]	T0, T15
Body fat percentage	BIA (Biostat Quadscan 4000, Douglas, Isle of Man)	T0, T3, T6, T15
Muscle mass	BIA (Biostat Quadscan 4000, Douglas, Isle of Man)	T0, T3, T6, T15
	24-h creatine excretion [44, 78]	T0, T3, T6, T15
Cardiometabolic risk factors		
Blood pressure	(Speidel & Keller Maxi Stabil 3)	T0, T3, T6, T15
Blood lipids	TG, TC, HDL-C, LDL-C in fasting serum levels (Cholesterol oxidase-phenol aminophenazone method, MEGA AU 510; Merck Diagnostica, Darmstadt, Germany)	T0, T3, T6, T15
Glucose metabolism		
Insulin resistance	HOMA-IR (fasting glucose, insulin)	T0, T3, T15 (all)
(Postprandial) hyperglycemia	7-point oral glucose tolerance test [47]	T0, T3, T15 (subgroup)
Renal parameters		
Medical history	Patient files	T0
Renal function	eGFR, albuminuria	T0, T3, T6, T15
Medical history	Patients files	T0, T3, T6, T15
Medication		
Immunosuppressants	Patient files, plasma drug levels	T0, T3, T6, T15
Other (e.g anti-hypertensives, anti-diabetics, statins)	Patient files	T0, T3, T6, T15
Psychological dimensions of lifestyle behavior		
Autonomy, competence, relatedness in general	Basic Needs Satisfaction in General Scale (BNSG [48, 49])	T0
Autonomy, competence, relatedness in exercise	Basic Psychological Needs in Exercise Scale (BPNES [50])*	T3, T6, T15
Self-regulation of exercise	Behavioral Regulation in Exercise Questionnaire (BREQ-2 [51])	T0, T3, T6, T15
Self-regulation and autonomy	Treatment Self-Regulation Questionnaire (TSRQ [52])	T3, T6
Motivation for exercise	Intrinsic Motivation Inventory (IMI [53]) adapted for exercise.	T3, T15
Motives for exercise	Exercise Motivation Inventory (EMI-2 [54])	T0, T6
Perceived competence	Perceived Competence Scale (PCS [56, 55])	T3, T6, T15
Perceived autonomy support	Health Care Climate Questionnaire (HCCQ [55, 57])*	T3, T6, T15
Fear of movement	Tampa Scale of Kinesiphobia – DV [58]	T0, T3, T15
Other questionnaires		
Demographics	Date of birth, marital status	T0, T3, T6, T15
	Sex, education, ethnicity	T0
Coping style	Utrecht Coping List [59]	T0
Nutrition knowledge	Based on questions of national dietary guidelines survey	T0, T15
Work-induced fatigue	Need for Recovery Scale (NFR [66])	T0, T3, T6, T15
Chronic fatigue	Dutch Checklist Individual Resilience (CIS-20 [60–62])	T0, T3, T6, T15
Work participation	NIVEL questionnaire [68]	T0, T3, T6, T15
Dietary intake		
Self-reported intake	3-day food diary	T0, T3, T6, T15
Intake biomarkers	24-h excretion of sodium, potassium, urea	T0, T3, T6, T15
Smoking and alcohol consumption	Questionnaire	T0, T6, T15
Process evaluation	Questions about tools, coaches and the program for the patient* Questions about tools, time/number of visits, and the program for the professional Focus groups with patients* Focus group with professionals	T15
Cost-effectiveness		
Quality of life	EQ-5D [69]	T0, T3, T6, T15
Care consumption, intervention costs	Standard Dutch Questionnaire	T0, T3, T6, T9, T12, T15

* These outcomes were only assessed in the intervention groups. Abbreviations: T#, months after baseline

### Self-reported physical functioning and quality of life

The physical functioning subscale of the short form 36 (SF-36), version 2 is used as a measure of physical functioning in the present study. Besides subjective measures of physical functioning, objective assessments are also included in the study as described below.

Furthermore, Quality of life is measured by the SF-36, the Kidney Disease Quality of Life Instrument - Short Form (KDQOL-SF) and the EuroQol (EQ-5D). SF-36 consists of 36 items which measure self-reported physical functioning, social functioning, role limitations due to physical and emotional problems, mental health, vitality, pain and general health on a scale of 0–100. The scores are added up into two summary scores, the physical component summary (PCS) and the mental component summary (MCS). The KDQOL-SF contains 13 items targeted at quality of life related to matters of special relevance for renal patients, such as disease burden and social interaction [38]. The KDQOL-SF has been translated into Dutch and validated in Dutch patient population [39]. The EQ-5D is a general questionnaire and contains questions on mobility, self-care, usual activities, pain/discomfort and anxiety/depression and a VAS scale for self-rated health.

### Objective measures of physical functioning

#### Muscle strength

Maximal muscle strength is assessed for multiple muscle groups (Additional file 1: Table S1). Maximal muscle strength of knee extensors, knee flexors, back and chest muscles are included as an outcome measure. Maximal muscle strength is determined using the one-repetition maximum (1RM) method under supervision of a qualified physiotherapist. The 1RM refers to the resistance at which the participant is only just able to perform the exercise correctly. For each strength test, the physiotherapist estimates the 1RM of the subject for the particular exercise machine. The increments are chosen in such a way that the participant reaches 1RM after 3–5 one-repetition sets.

#### Aerobic capacity

Aerobic capacity is determined by symptom-limited graded cycle ergometry, which is commonly used in renal transplant patients and considered the gold standard for determining aerobic capacity [1]. The test determines the maximal workload (Wpeak) and maximal oxygen uptake (VO_2_peak) using a respiratory gas analyzer (Cortex Metalyzer 3B breath analyser, Cortex Biophysik GmbH, Leipzig, Germany). VO_2_peak indicates the peak amount of oxygen that the body is able to utilize during sustained physical exertion [1]. The cycle ergometers used for all participants is a Lode Corival 906900 (Lode B.V., Groningen, Netherlands). After a period of rest and 1 min pedaling without workload, the bicycle workload is increased gradually every minute by 10 to 25 W depending on the participants’ expected exercise capacity. The incremental increase in workload is done in such a way that maximal exercise capacity is reached within 8–12 min after starting the exercise test. The workload during the test is blinded for the participant. Participants cycle at a cadence of 60–70 rotations per minute until exhaustion. The maximal cycle ergometry tests are medically supervised by a sports physician. Before and during the test, the participant’s heart function is monitored using ECG-monitoring. In addition, heart rate, blood pressure and oxygen saturation are monitored. The test is stopped when ECG abnormalities occur, systolic blood pressure rises over 250 mmHg or diastolic blood pressure rises over 120 mmHg, or if blood pressure falls with more than 20 mmHg.

The sub-maximal exercise test is performed on a general fitness cycle-ergometer under supervision of a qualified physiotherapist. It consists of a 2 min warm-up, 10 min cycling at 50% Wpeak, followed by 70% of Wpeak at 60–70 rpm until exhaustion, but for a maximum of 20 min. A longer test duration indicates better exercise capacity. Heart rate and blood pressure are monitored during the submaximal cycle ergometry using an Omron M6 (Omron, Tokyo, Japan).

#### Balance and mobility

The Short Physical Performance Battery (SPPB) is used to determine lower extremity function as a measure for balance and mobility. SPPB is a common and reliable standardized measure of balance, gait speed, and chair stand tests used to measure physical performance [40, 41].

For daily physical activity, the Baecke questionnaire is used to assess habitual PA with questions on PA in different domains such as school/work, sports practice and leisure-time physical activity [42]. Responses are combined producing an overall habitual activity score. This has been validated against the doubly-labeled water method, showing a strong correlation (*r* = 0.69, p = <0.001) [43]. A validated spring-levered step counter (Yamax Digiwalker SW-200, Lees Summit, Missouri (YX200)) is used to objectively measure daily steps as an objective measure of physical activity level. Participants are instructed to wear the step counter for 5 days, and record the date, number of steps at each of the five days, as well as the time in minutes spent on cycling, swimming and fitness on each of those 5 days.

### Body composition and nutrition status

Body composition is estimated using a multi-frequency bio-electrical impedance device (BIA, Biostat Quadscan 4000, Douglas, Isle of Man) at 5, 50, 100 and 200 Hz, which allows for distinction between body fat mass and lean body mass taking into account differences in volume status. BIA is used to estimated fat mass, fat free mass and body fat percentage. Muscle mass is estimated as fat free mass from BIA and from 24-h urinary creatinine excretion. The 24-h creatinine excretion is considered a reliable measure of muscle mass even in patients with advanced renal failure, in elderly people, and in patients with wasting conditions [3, 44]. Anthropometry measures include bodyweight, height, waist- and hip circumference. All anthropometry measures are done twice. Bodyweight is measured in lightweight clothing without shoes, preferably in the morning, on a calibrated scale (Seca 877, Seca GMBH, Hamburg, Germany), to the nearest 0.1 kg. Height is measured using a wall-secured stadiometer (Seca 222, Seca GMBH, Hamburg, Germany). Waist circumference is measured with the subject in standing position at the level midway between the lowest rib and iliac crest at the end of an expiration to the nearest 0.1 cm. Waist circumference is also measured in participants with polycystic kidney disease which may create noise in the data, although in case a participant had polycystic kidney disease this is clearly defined in our dataset. Hip circumference is measured as the maximum circumference over the trochanter major to the nearest 0.1 cm. A tape with standardized retraction mechanism (Seca 201, Seca GMBH, Hamburg, Germany) is used. Nutrition status is assessed by subjective global assessment (SGA) [45]. The SGA consists of an anamnesis (questions about change in bodyweight, food intake and gastrointestinal symptoms) and a physical examination (subcutaneous fat, muscular atrophy of the upper and lower body) resulting in an SGA-classification (seriously malnourished, moderately malnourished or normally nourished).

### Cardiometabolic risk factors

Blood samples are collected after an overnight fast and assayed for triglycerides, total cholesterol, HDL- and LDL cholesterol for lipid profile and for glucose and insulin to calculate insulin resistance (HOMA2-index) [46]. Impaired glucose metabolism is measured using 7-timepoint Oral Glucose Tolerance Test [47]. After fasting blood sampling, the participant consumes a 300 ml glucose solution which contains 75 g of glucose (82.5 g glucose monohydrate). After glucose ingestion, blood is drawn at 10, 20, 30, 40, 60, 90 and 120 min. During the test participants are not allowed to eat or drink, except for a limited amount of water. Blood samples are stored until further analysis. Oral Glucose Tolerance Testing was performed in a subsample (participants from University Medical Center Groningen).

### Renal parameters

Serum creatinine concentrations are determined using the Jaffé method. Creatinine clearance is calculated from 24-h urinary creatinine excretion and serum creatinine. Medical information is collected from the participants’ medical files, including renal parameters (underlying cause of the renal disease, dialysis duration and type, transplantation type, time since transplantation), medication use, hospital admissions, complications after transplantation, co-morbidities and mortality. Medication is derived from the participants’ medical records.

### Questionnaires

A quite extensive set of questionnaires to address potential psychological aspects, mostly aspects of motivation, is included in the questionnaires for participants. Details about moment of assessment is given in Table 1. The following questionnaires are included: Basic Needs Satisfaction in General Scale [48, 49]Basic Psychological Needs in Exercise Scale [50], Behavioral Regulation in Exercise Questionnaire version 2 [51], Treatment Self-regulation Questionnaire [52], Intrinsic Motivation Inventory [53], Exercise Motivations Inventory version 2 [54], Perceived Competence Scale [55, 56] and the Health Care Climate Questionnaire [55, 57]. Furthermore, fear of movement is determined using the 17-item Tampa Score for Kinesiophobia [58]. Questions are answered on a 4-point likert-type scale ranging from 0 (not true for me) to 4 (very true for me). Coping style is measured using the Utrecht Coping List, a Dutch 47-item questionnaire [59]. Coping style refers to a persons’ specific efforts to master, tolerate, or reduce stress in stressful conditions. Nutrition knowledge is estimated using items from a national survey, developed by the Netherlands Nutrition Center to assess knowledge of the Dutch dietary guidelines on dietary fibre, fruits, vegetables, calories, fats, and extended with items about potassium and sodium. Chronic fatigue is measured using the Dutch Checklist Individual Resilience (CIS-20) [60–62] consisting of 20 items covering the subjective experience of fatigue, reduction in motivation, reduction in concentration and reduction in physical activity. CIS-20 is tested thoroughly in the clinical setting among patients with chronic fatigue syndrome and other chronic diseases and healthy controls [63–65]. Work-induced fatigue is assessed by the Need for Recovery scale [66]. This 11-item questionnaire measures fatigue as a result of exposure to physical and mental demands imposed by work [66, 67]. Work participation is assessed using a previously developed questionnaire for patients with renal disease [68]. Furthermore, the ACT questionnaire contains questions about demographics, smoking, and alcohol consumption.

### Dietary intake

A three-day food diary is kept for two weekdays and one weekend day. Participants are instructed to fill out the food diary accurately using standard supply units or, in case of deviations, to weigh their food to improve estimate of consumption. Food diaries are analyzed by a dietitian, and the nutrition values are calculated using the software program EvryDietist, 6.4.2.1 (Evry, Alphen aan den Rijn, Netherlands), using the Dutch Food Composition Database 2016 to calculate macro- and micronutrient intake. As part of usual care all participants are instructed to collect a 24-h urine sample according to a strict protocol at the day before their visit to the outpatient clinic, from which dietary sodium intake is calculated. Twenty-four-hour urine collections are stored until further analysis.

### Process evaluation

Qualitative findings regarding organizing and carrying out the intervention is derived from focus groups with the participants, and separately with 1 focus group with professionals (physiotherapists, dieticians and lifestyle coaches). All participants that are randomized into the intervention groups are invited to take part in the focus group after they have finished the intervention. Each participant focus group lasts 90 min, during which they are asked a series of open questions to evaluate the exercise training phase, the follow-up phase, the lifestyle counseling sessions and dietary counseling sessions. The focus groups are recorded and the recordings are transcribed for analysis. A focus group with the professionals will be done at the end of the ACT study.

### Health economic analysis

Costs en cost effectiveness of the interventions will be studied from a societal perspective over a 15 month time horizon. The effectiveness of the interventions will be assessed using information about the health-related quality of life of the participants, measured using the EQ-5D [69]. This instrument measures health-related quality of life and 60 translates responses into a utility score. The EQ-5D measures health in five dimensions of health-related quality of life (mobility, self-care, usual activities, pain/discomfort, and anxiety/depression). Dutch-specific value sets will be used to translate responses into a utility score. Utility scores will be used to calculate quality-adjusted life-years (QALYs).

Healthcare consumption is measured using a standardized Dutch questionnaire. This questionnaire measures the amount of visits to a healthcare provider, and the means of travelling (i.e. walking/cycling, car, public transport, taxi or other). Unit costs from routinely available sources will be applied to the number of visits to calculate the total costs of these visits for each participant. The Dutch manual for cost-effectiveness analysis is used as a guideline for weighing the cost items [70, 71]. Additional cost calculations will be done for the interventions including personnel, materials, volume and overhead costs. The total costs and QALYs in the intervention groups will be compared to the total costs and QALYs in the control group. An incremental cost-effectiveness ratio (ICER) will be calculated to compare the exercise intervention, and the exercise + diet intervention with usual care. Furthermore, two cost-utility analyses will be done with EQ-5D as an outcome measure.

### Statistical analyses

Short term differences between groups will be tested using students T-test or linear regression, and long term differences will be tested using General Linear Mixed models. Time will be analyzed for exercise intervention (phase 1) and maintenance period (phase 2). Centers will be taken into account as random factor. Intention to treat principle will be applied in primary analyses. Per protocol analysis will be based on completion of the exercise training sessions, and attendance of dietary counseling sessions. Stratified analysis are foreseen for time after transplantation, socio-economic status and immunosuppressive regime. Furthermore, potential preset determinants of intervention outcome will be evaluated in a GLM model: gender, age, coping style, fear of movement, work participation and clinical factors.

## Discussion

The aim of this RCT is to evaluate the effects and cost-effectiveness of an exercise only intervention and an exercise + diet intervention on physical functioning, quality of life and -as an important secondary aim- post-transplantation weight gain and cardiometabolic risk after renal transplantation. It will cover a large range of relevant patient-centered and clinical outcomes, as well as cost-effectiveness analyses, to provide guidance for evidence-based care.

The ACT trial is a pragmatic RCT. This means that already in the trial phase, the intervention is aimed at and implemented by regular staff members in daily care. This also implies that the control condition can be less controlled than it would have been in an explanatory trial [24, 25].

In the present study we strive for a set-up to maximize external validity within the context of renal transplant care in the Netherlands, by working with physiotherapists in existing rehabilitation centers and physiotherapy practices, and with dieticians in the field of renal care. Hereby generalizability to the clinical setting in RTR population is offered, providing widely applicable high-quality evidence for healthcare policy makers. Evidence-based behavioral components are included in the study to facilitate behavior change and long-term behavior maintenance.

Another strength is the application of focus groups with both the participants as well as the various healthcare professionals, as these qualitative data will provide a wealth of detailed information of patients’ and professionals’ attitudes toward and experiences with the intervention, supporting future implementation.

The present RCT includes a relatively high number of participants. The exercise training is delivered in larger groups at rehabilitation centers, and in smaller groups with physiotherapy practices. Sub-analyses may reveal differences in outcome between settings. The diet intervention is done by renal dieticians which are familiar with this patient population. Other strengths include gold standard measurement of aerobic capacity, objective measurement of physical activity using step counters, bio-impedance and BMI measured by dieticians, and the use of 24-h urine biomarkers.

A limitation of the present study is its complexity. The RCT involves skills and experience of multiple healthcare professionals working at different centers, making it difficult to standardize intervention delivery. Another limitation is that the exercise intervention and exercise with dietary counseling groups are combined. This may lead to contamination between groups, as participants of the exercise + diet group may share dietary advice with the exercise only group e.g. during the exercise training sessions. Furthermore, participants entering the ACT trial already have a certain degree of motivation to improve their health and fitness through lifestyle. Therefore, control participants may have worked on their health and fitness in alternative ways. Another limitation is that the study took place only in the Netherlands.

The present study aims to provide evidence needed to guide decision making by clinicians and policy-makers in post-transplantation care. Furthermore, it will help develop guidelines for post-transplantation care in the Netherlands to further improve long-term outcomes of renal transplantation.

## Additional files

Additional file 1: Table S1. Overview of resistance exercises. (DOCX 25 kb)

Additional file 2: Table S2. Exercise protocol of the ACT trial. (DOCX 26 kb)

## Abbreviations

1RM: One repetition maximum
ACT: Active care after transplantation
BIA: Bio-impedance analyzer
BMI: Body mass index
CIS-20: Checklist individual strength
CONSORT: Consolidated standards of reporting trials
EQ-5D: EuroQOL 5 dimensions
HbA1c: Glucose-bound haemoglobin
KDQOL: Kidney disease quality of life
KG: Kilograms
Pmax: Maximal muscle strength
QALY: Quality-adjusted life year
RCT: Randomized controlled trial
RTR: Renal transplant recipients
SF-36: Short Form 36
SGA: Subjective global assessment
SPPB: Short physical performance battery
VO_2_peak: Peak Oxygen uptake, a measure for aerobic capacity
Wpeak: Peak load (Watts), as assessed during the symptom-limited cycle ergometry
