# Supplementary material for: Lifestyle intervention to improve quality of life and prevent weight gain after renal transplantation: Design of the Active Care after Transplantation (ACT) randomized controlled trial
Source: BMC Nephrol. 2017 Sep 15;18:296. doi: 10.1186/s12882-017-0709-0 (PMC5599936; doi:10.1186/s12882-017-0709-0)
Supplement: Supplementary file 1 — Overview of resistance exercises. (DOCX 25 kb) [file 12882_2017_709_MOESM1_ESM.docx]

**Additional file 1: Table S1.** Overview of resistance exercises

| **Muscle group** | **Method** | **Unit** |
| --- | --- | --- |
| Quadriceps femoris | Leg extension* | 1RM (Kg) |
| Semimenbranosus, semitendinosus, biceps femoris | Leg curl* | 1RM (Kg) |
| Biceps brachii, latissimus dorsi, teres major | Close-grip pull-down* | 1RM (Kg) |
| Biceps brachii, latissimus dorsi, teres major | Lateral pull-down | 1RM (Kg) |
| Pectoralis major, deltoideus anterior, triceps brachii | Chest press* | 1RM (Kg) |
| Pectoralis major en deltoideus anterior | Pectoral machine | 1RM (Kg) |
| Trapezius, deltoideus posterior, subscapularis | Rowing Torso | 1RM (Kg) |
| Rectus abdominis, obliquus abdominis | Abdominal Crunch | 1RM (Kg) |

*Included in secondary study outcomes
