# Supplementary material for: Lifestyle intervention to improve quality of life and prevent weight gain after renal transplantation: Design of the Active Care after Transplantation (ACT) randomized controlled trial
Source: BMC Nephrol. 2017 Sep 15;18:296. doi: 10.1186/s12882-017-0709-0 (PMC5599936; doi:10.1186/s12882-017-0709-0)
Supplement: Supplementary file 2 — Exercise protocol of the ACT trial. (DOCX 26 kb) [file 12882_2017_709_MOESM2_ESM.docx]

|  | Resistance exercise | Aerobic exercise |
| --- | --- | --- |
| Week 1, session 1 | **Test session 1 Test session 1** | |
| Week 1, session 2 | 1 set / 30 repetitions  25% Pmax 1 | 10 min cycling (50% Wmax 1)  10 min walking* (HR = HR cycling) |
| Week 2, session 1 | 2 sets / 8 repetitions  50% Pmax 1 | 12 min cycling (50% Wmax 1)  12 min walking (HR = HR cycling) |
| Week 2, session 2 | 2 sets / 30 repetitions  25% Pmax 1 | 12 min cycling (50% Wmax 1)  12 min walking (HR = HR fiets training) |
| Week 3, session 1 | 2 set / 10 repetitions  50% Pmax 1 | 14 min cycling (50% Wmax 1)  14 min walking (HR = HR cycling) |
| Week 3, session 2 | 2 sets / 30 repetitions  30% Pmax 1 | 14 min cycling (50% Wmax 1)  14 min walking (HR = HR cycling) |
| Week 4, session 1 | 2 sets / 10 repetitions  60% Pmax 1 | 14 min cycling (60% Wmax 1)  14 min walking (HR = HR cycling) |
| Week 4, session 2 | 2 sets / 30 repetitions  30% Pmax 1 | 14 min cycling (60% Wmax 1)  14 min walking (HR = HR cycling) |
| Week 5, session 1 | 2 set / 10 repetitions  60% Pmax 1 | 14 min cycling (65% Wmax 1)  14 min walking (HR = HR cycling) |
| Week 5, session 2 | 2 sets / 30 repetitions  35% Pmax 1 | 14 min cycling (65% Wmax 1)  14 min walking (HR = HR cycling) |
| Week 6, session 1 | 2 sets / 10 repetitions  60% Pmax 1 | 14 min cycling (70% Wmax 1)  14 min walking (HR = HR cycling) |
| Week 6, session 2 | **Test session 2** | 10 min cycling (50% Wmax1)  10 min walking (HR = HR cycling) |
| Week 7, session 1 | 2 set / 10 repetitions  50% Pmax 2 | 14 min cycling (60% Wmax 1)  14 min walking (HR = HR cycling) |
| Week 7, session 2 | 2 sets / 30 repetitions  25% Pmax 2 | 14 min cycling (65% Wmax 1)  14 min walking (HR = HR cycling) |
| Week 8, session 1 | 3 sets / 10 repetitions  50% Pmax 2 | 14 min cycling (65% Wmax 1)  14 min walking (HR = HR cycling) |
| Week 8, session 2 | 3 set / 30 repetitions  25% Pmax 2 | 14 min cycling (65% Wmax 1)  14 min walking (HR = HR cycling) |
| Week 9, session 1 | 3 sets / 10 repetitions  50% Pmax 2 | 14 min cycling (70% Wmax 1)  14 min walking (HR = HR cycling) |
| Week 9, session 2 | 3 sets / 30 repetitions  30% Pmax 2 | 14 min cycling (70% Wmax 1)  14 min walking (HR = HR cycling) |
| Week 10, session 1 | 3 sets / 10 repetitions  60% Pmax 2 | 14 min cycling (70% Wmax 1)  14 min walking (HR = HR cycling) |
| Week 10, session 2 | 3 set / 30 repetitions  30% Pmax 2 | 14 min cycling (75% Wmax 1)  14 min walking (HR = HR cycling) |
| Week 11, session 1 | 3 sets / 10 repetitions  60% Pmax 2 | 14 min cycling (75% Wmax 1)  14 min walking (HR = HR cycling) |
| Week 11, session 2 | 3 sets / 30 repetitions  35% Pmax 2 | 14 min cycling (80% Wmax 1)  14 min walking (HR = HR cycling) |
| Week 12, session 1 | 3 sets / 10 repetitions  60% Pmax 2 | 14 min cycling (80% Wmax 1)  14 min walking (HR = HR cycling) |
| Week 12, session 2 | **Test session 3** | **Test session 3** |

**Additional file 2: Table S2.** Exercise protocol of the ACT trial

*Pmax 1= maximal strength at test session 1 (baseline); Pmax2= maximal strength at test session 2; HR= heart rate;
Wmax1= maximal load based on symptom-limited graded cycle ergometry (baseline)*

*Test session 1: maximal strength tests + submaximal cycle ergometry*

*Test session 2: maximal strength tests + light intensity aerobic exercise as a recovery session*

*Test session 3: maximal strength tests + submaximal cycle ergometry*
